# Relapse of Acute Myeloid Leukemia With Concomitant Systemic Mastocytosis Five Years Post Allogenic Hematopoietic Stem Cell Transplantation

**DOI:** 10.1155/crh/8965147

**Published:** 2026-01-30

**Authors:** Sona Vardanyan, Hilde K. Gjelberg, Lars Helgeland, Galina Tsykunova, Rakel Brendsdal Forthun, Håkon Reikvam

**Affiliations:** ^1^ Department of Oncology, Haukeland University Hospital, Bergen, Norway, helse-bergen.no; ^2^ Department of Pathology, Haukeland University Hospital, Bergen, Norway, helse-bergen.no; ^3^ K.G. Jebsen Center for Myeloid Blood Cancer, Department of Clinical Science, Norway Department of Clinical Science, University of Bergen, Bergen, Norway, uib.no; ^4^ Department of Medical Science, University of Bergen, Bergen, Norway, uib.no; ^5^ Department of Medicine, Haukeland University Hospital, Bergen, Norway, helse-bergen.no; ^6^ Cancer Genomics, Haukeland University Hospital, Bergen, Norway, helse-bergen.no

**Keywords:** acute myeloid leukemia, allogenic hematopoietic stem cell transplantation, clonal evolution, relapse, systemic mastocytosis

## Abstract

Systemic mastocytosis (SM) with associated hematological neoplasia (SM‐AHN) is a rare and aggressive condition characterized by abnormal clonal proliferation of mast cells and the concurrent occurrence of hematologic malignancies, such as acute myeloid leukemia (AML). We present a 41‐year‐old female diagnosed with SM‐AML, who underwent allogeneic hematopoietic stem cell transplantation (allo‐HSCT). Despite an initial favorable response to chemotherapy and transplantation, the patient later experienced an AML relapse five years post‐transplant, without concurrent recurrence of SM. This discrepancy may be attributed to the differential immune responses to AML and SM, where AML cells are more susceptible to graft‐versus‐leukemia (GVL) effects, while mast cells in SM may exhibit resistance to immune‐mediated elimination. The absence of SM relapse raises important questions regarding the pathophysiology and treatment of SM‐AML. This case underscores the complexity of managing SM with AML, highlighting the need for further research to optimize therapeutic strategies and improve patient outcomes.

## 1. Introduction

Systemic mastocytosis (SM), a subgroup of mastocytosis defined by the World Health Organization (WHO), is a highly heterogenous disease characterized by its abnormal clonal mast cell proliferation in one or more extracutaneous organs [1–3]. Bone marrow involvement is a major criterion, whereas minor criteria include the presence of CD25 cells [1, 2]. In most cases of SM, a characteristic finding is the presence of *KIT* (CD117) mutations [2, 4], leading to increased tyrosine kinase activity and thus promotion of clonal proliferation and progression [4]. The WHO has further classified SM with associated hematological neoplasm (SM‐AHN) [2, 5]. Among these is SM‐AML with acute myeloid leukemia (AML).

AML is a highly aggressive bone marrow malignancy characterized by the rapid proliferation and accumulation of immature myeloid leukemic blasts [6]. The malignancy is defined by the presence of at least 20% immature undifferentiated leukemic blast cells in the bone marrow or specific genetic mutations [6, 7]. Although *KIT* mutations are present in some AML cases, especially in core binding factor (CBF)‐AML, the synchronous development of SM is rather rare, suggesting that other specific genetic events must occur for the development of concurrent SM [8].

SM‐AML is classified as an aggressive disease, with the primary treatment focus on targeting the leukemia component, typically through allogeneic hematopoietic stem cell transplantation (allo‐HSCT) [5, 8]. However, clinical knowledge of this is limited, and information is restricted to a few case reports or serial cases [8].

## 2. Case Presentation

**FIGURE 1:**
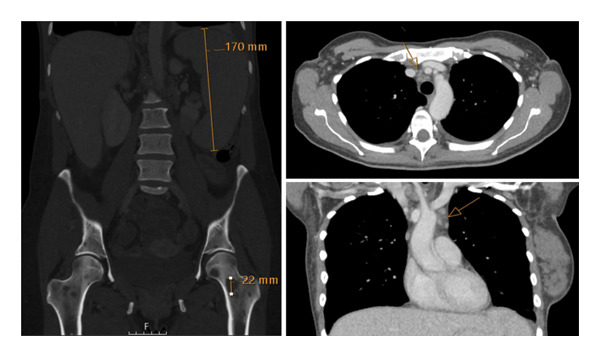
CT scan at the time of diagnosis, showing an enlarged spleen (17 cm), and multiple osteolytic lesions, the largest in the neck of the left femur (22 mm).

**FIGURE 2:**
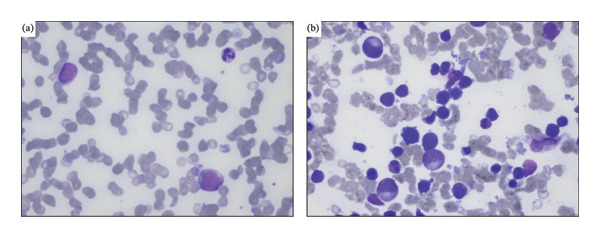
(a) Peripheral blood and (b) bone marrow smear at the time of diagnosis. The peripheral blood smear shows two myeloid blast cells and one mature neutrophil. The bone marrow smear demonstrates immature myeloid cells (large cells), dysplastic erythroid cells, and eosinophils. No mast cell aggregates were seen.

**FIGURE 3:**
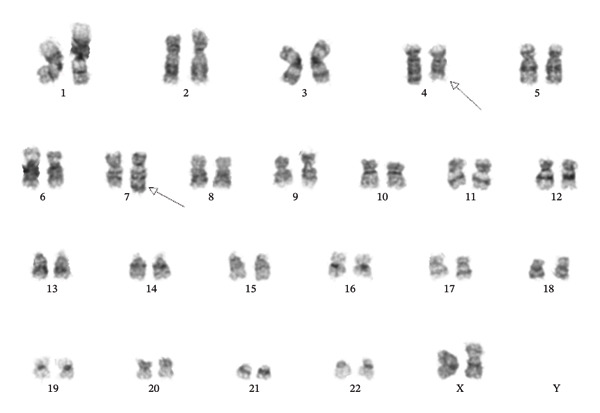
G‐banding analysis in 20 metaphases showing a balanced translocation between chromosome 4 band q31.1 and chromosome 7 band, (46,XX,t(4; 7)(q31.1; q36)).

**FIGURE 4:**
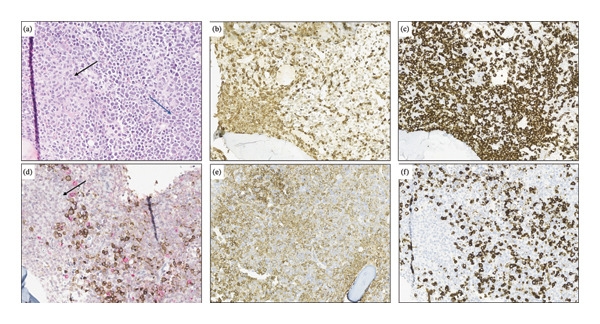
(a) Bone marrow histology at the time of diagnosis, demonstrating aggregates of atypical mast cells (black arrow) and irregular hematopoiesis with immature myeloid cells and myeloid blast cells (blue arrow) (H&E, original magnification 32x). (b) Immunohistochemical staining for mast cell tryptase demonstrating aggregates of mast cells. (c) The mast cells show aberrant expression for CD25. (d) CD71 and lysozyme demonstrating scattered erythroid cells (brown). The blasts are partly lysozyme positive (red). The mast cells are negative (arrow). Both mast cells and myeloid blasts demonstrate diffuse positivity for CD117 (e). The myeloid blast cells are positive for CD34 (f) (B‐C: Original magnification 20x, D‐F: B‐C: Original magnification 25x).

A 41‐year‐old woman was referred to hospital with tachypnea, tachycardia, and hypoxemia. Her laboratory workup revealed pancytopenia (Table 1) and slightly elevated alkaline phosphatase (ALP), gamma‐glutamyl transferase (GGT), and lactate dehydrogenase (LDH) (Table 1). A CT scan revealed no liver pathology; however, spleen enlargement measuring 17 cm in length was found, in addition to multiple enlarged lymph nodes in the mediastinum (Figure 1). Additionally, CT uncovered bilateral pleural effusion and multiple osteolytic lesions; the largest was found in the neck of the left femur with a diameter of 22 mm (Figure 1). Examination of peripheral blood smear revealed a homogenous blast population of 18% with round nuclei and clear nucleoli, without Auer rods (Figure 2). This prompted further investigation with a bone marrow examination, displaying a bone marrow smear with 78% immature myeloid and blast cells with little to no cytoplasm (Figure 2). Immunophenotyping showed a myeloblastic phenotype (Table 2), leading to the diagnosis of AML, classified as FAB M0. G‐banding analysis revealed a balanced translocation between chromosome 4 and chromosome 7; (46,XX,t(4; 7)(q31.1; q36)) (Figure 3). Next generation sequencing (NGS) myeloid panel unveiled the following mutations: *RUNX1* p.(Thr111_Leu112del) (variant allele fraction (VAF) 39.4%), *RUNX1* p.(Asp198Asn) (VAF 34.4%), *BCOR* p.(Pro1062Glnfs∗51) (VAF 35.2%), *ASXL1* p.(Gly646Trpfs∗12) (VAF 26.8%), and *KIT* p.(Asp816Val) (VAF 21.5%) (Table 2). The bone marrow biopsy showed 100% cellularity, with multiple aggregates of atypical epithelioid to spindle mast cells, demonstrating positivity for mast cell markers CD117 (CKIT) and mast cell tryptase and aberrant expression for CD25 by immunohistochemistry. The mast cells accounted for 30% of the cells in the bone marrow, confirming at least one C‐finding, alongside splenomegaly on CT and cytopenia, both of which, however, could have been part of the AML. The remaining hematopoiesis showed an expanded and left‐shifted myelopoiesis with a large population of blast cells constituting approximately 30%–40%. Otherwise, dysplastic erythropoiesis and small, irregular megakaryocytes were present. The findings were consistent with systemic mastocytosis with associated hematological neoplasm (Figure 4).

**TABLE 1:** Laboratory workout at primary diagnosis and relapse.

Measurements	References	Primary diagnosis	Relapse
Hemoglobin	11.7–14.3 g/dL	8.6 g/dL	7.8 g/dL
WBC	3.5–11.0 × 10^9^/L	2.7 × 10^9^/L	21.1 × 10^9^/L
ANC	1.7–8.2 × 10^9^/L	0.4 × 10^9^/L	0.1 × 10^9^/L
Platelet count	165–387 × 10^9^/L	45 × 10^9^/L	23 × 10^9^/L
ALP	35–105 U/L	216 U/L	93 U/L
GGT	10–75 U/L	147 U/L	128 U/L
LDH	105–205 U/L	282 U/L	318 U/L
Tryptase	< 12 μg/L	281 μg/L	5.9 μg/L

*Note:* ALP: alkaline phosphatase; LDH: lactate dehydrogenase; g: gram, μg: microgram; dL: deciliter, L: liter.

Abbreviations: ANC: absolute neutrophil count; GGT: gamma‐glutamyl transpeptidase; WBC: white blood cells.

**TABLE 2:** Immunophenotypic and mutational features of leukemic blasts at diagnosis and at relapse.

Gene	Variant	NM‐number	VAF (primary diagnosis)	VAF (relapse)
RUNX1	p.(Thr111_Leu112del)	NM_001754.5	39.4%	41.5%
RUNX1	p.(Asp198Asn)	NM_001754.5	34.4%	38.4%
BCOR	p.(Pro1062Glnfs∗51)	NM_001123385.2	35.2%	40.2%
ASXL1	p.(Gly646Trpfs∗12)	NM_015338.6	26.8%	35.0%
KIT	p.(Asp816Val)	NM_000222.2	21.5%	N.D.
KRAS	p.(Gln61His)	NM_004985.5	N.D.	5.9%
KRAS	p.(Gly12Val)	NM_004985.5	N.D.	5.7%
NRAS	p.(Gly12Val)	NM_002524.5	N.D.	35.0%
NRAS	p.(Gly12Cys)	NM_002524.5	N.D.	11.7%

*Note:* The table summarizes the key molecular mutations and surface marker expression at the time of diagnosis and relapse. For mutational status, percentages represent variant allele frequency (VAF), while for surface markers, percentages indicate the proportion of positive cells.Abbreviation: N.D = not detected.

The diagnosis was confirmed with the presence of B‐findings such as elevated serum tryptase of 281 µg/L and alkaline phosphatase of 216 U/L (Table 1), in addition to C‐findings [3]. The patient was cachectic and had experienced symptoms for about six months before the diagnosis. No cutaneous signs of mastocytosis, such as urticaria pigmentosa, were observed at any point. Dermatological assessment was negative at diagnosis and during follow‐up.

Four days after hospitalization, induction chemotherapy with idarubicin 12 mg/m^2^ (Days 1–3), and cytarabine 200 mg/m^2^ (Days 3–7) was initialized, and midostaurin 50 mg twice daily was added from Day +8. On Day +21, a new bone marrow biopsy was performed with the presence of about 30% mast cells and 20%–30% immature blast cells. Due to an incomplete remission status, the patient started a second induction chemotherapy regimen with cytarabine 2000 mg/m^2^ (Days 1–6), daunorubicin 60 mg/m^2^ (Days 1, 3, and 5), and midostaurin 50 mg twice daily from Day +8. On Day +17, the examination of bone marrow confirmed complete hematological remission (1% blast cells, 4% promyelocytes, and 4% lymphocytes). A small population of mast cells was present, compatible with residual infiltrates of SM.

The patient proceeded to allogenic hematopoietic stem cell transplantation (allo‐HSCT). She received myeloablative conditioning with fludarabine and busulfan, and antithymocyte globulin (ATG), and then underwent allo‐HSCT with bone marrow derived stem cells received from an unrelated 21‐year‐old female donor with 10/10 (11/12) HLA‐match, resulting in hematological engraftment on Day +23 after transplantation. She received standard graft versus host disease (GvHD) prophylaxis with cyclosporine A and methotrexate; however, they still developed acute graft versus host disease (aGvHD) in the skin on Day +52, grade 1/stage 1, and was treated with extracorporeal photopheresis (ECP) for four months. After three months (Day +90) post transplantation, the chimerism status was controlled, revealing the chimerism status at 97% in total, of which 74% T‐cells, granulocytes‐monocytes 99%. Furthermore, no *KIT* mutation was detected, and the tryptase level was normal. During regular checkup at six months after transplantation, the patient started treatment for a potential pulmonary GvHD/bronchitis obliterans syndrome (BOS) with and FEV1 of 52%, receiving this for almost 2 years. Nine months post transplantation, the patient presented with full (> 99%) donor chimera. It was concluded with complete remission of both AML and mastocytosis at follow‐up consultations one, two, three, and four years after allo‐HSCT. These included regular bone marrow biopsies for morphological evaluation, clinical and biochemical examinations, and follow‐up with pulmonary specialists regarding BOS. To summarize, during the first year of follow‐up, the patient experienced productive cough, a decline in FEV1 to 52%, and was treated with steroid inhalations, monteleukast, and ECP for the potential BOS, which led to a tremendous increase of FEV1 to > 80%. During her second year of follow‐up, she weaned off steroid inhalations, and during her third year of follow‐up, she was off steroids, with no signs of GvHD and examinations showed complete remission and normal tryptase levels. Due to hypogammaglobulinemia, she was treated with intravenous immunoglobulin, which was reversed by the 4th year of follow‐up. She did report episodes of fatigue although exhibited a Karnofsky score of 90%.

**FIGURE 5:**
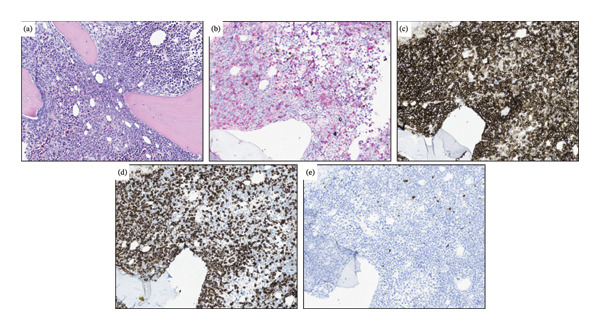
(a) Bone marrow histology at the time of relapse demonstrating a uniform proliferation of myeloblasts that replace the pre‐existing hematopoiesis (H&E, original magnification 25x). (b) Immunohistochemical staining for CD71 and lysozyme demonstrating only few scattered erythroid cells (brown). The blasts are partly lysozyme positive (red). The blast cells demonstrate diffuse positivity for CD117 (c) and are partially positive for CD34 (d). Only few scattered mast cells are seen in staining for mast cell tryptase (e) (B–E: Original magnification 16x).

Five years postinitial diagnosis, the patient reported several months of fatigue, followed by a one‐month course of COVID‐19 infection. She presented with diffuse petechiae on the thighs and legs. Laboratory evaluation demonstrated anemia, thrombocytopenia, and leukocytosis (Table 1). Relapse of the disease was suggested, and bone marrow examinations were performed. Flow cytometry of bone marrow displayed > 65% viable white cells were immature, and the immunophenotyping features compared with the initial diagnosis are demonstrated in Table 2. It was noted that the immature myeloid cells showed a rather heterogenous immunophenotype and that expanded cells could partly be described as two separate populations; CD34+ cells that were weaker in CD45 expression and had a stronger CD13 expression. Bone marrow biopsy confirmed the presence of increased levels of immature blast cells, 40% being CD34 positive, and 70% being CD117 positive (Figure 5). The bone marrow biopsy did not exhibit presence of mast cells (Figure 5), and serum tryptase levels were within the reference range (Table 1). These findings were consistent with a relapse of AML although without the coexisting SM that was present as part of the SM‐AML at the initial diagnosis.

**FIGURE 6:**
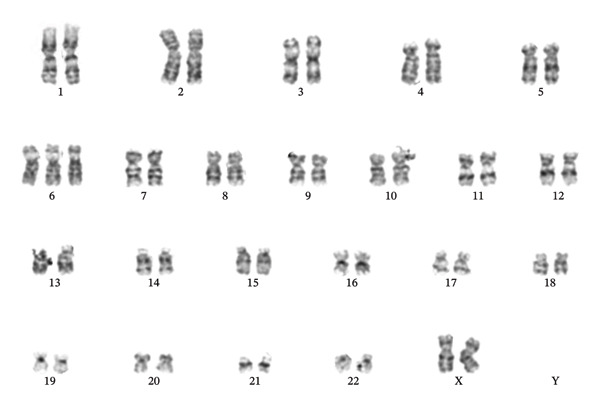
G‐banding at the point of relapse examined 20 metaphases, with findings of trisomy 6: 47, XX, +6.

Donor chimeric status was performed, demonstrating blood before cell separation of 33%, 97% T‐cell (CD2+), and all other cells (myeloid) (CD2‐) at 11%. Due to the low donor chimerism, the relapse was not considered to be of donor cell origin. G‐banding at the point of relapse showed trisomy 6; 47,XX,+6 (Figure 6), with no signs of the original translocation. NGS myeloid panel showed the following mutations (Table 2): *RUNX1* p.(Thr111_Leu112del) (VAF 41.5%), *RUNX1* p.(Asp198Asn) (VAF 38.4%), *BCOR* p.(Pro1062Glnfs∗51) (VAF 40.2%), *ASXL1* p.(Gly646Trpfs∗12) (VAF 35.0%), *KRAS* p.(Gln61His) (VAF 5.9%), *KRAS* p.(Gly12Val) (VAF 5.7%), *NRAS* p.(Gly12Val) (VAF 35.0%), and *NRAS* p.(Gly12Cys) (VAF 11.7%), although negative for *KIT* mutations (Table 2). *KIT* negativity was established by NGS analysis from the bone marrow.

Owing to the presentation of the relapse, it was considered a high‐risk disease. After treating the infection, reinduction chemotherapy with the FLAG‐Ida‐Ven regimen was initiated (Days 1–6: filgastrim 48 mil IE once daily, Days 2–6: fludarabine 30 mg/m^2^, Days 2–6: cytarabine 2000 mg/m^2^, Days 2–4: idarubicin 6 mg/m^2^, and Days 2–8: venetoclax 400 mg once daily), with complete hematological remission on Day +33 (regenerative marrow in remission with the presence of 1% blast cells).

The patient underwent a second allo‐HSCT, with blood derived stem cells from a matched unrelated donor. She was conditioned with fludarabine, treosulfan, and ATG. Bone marrow biopsy on Day +28 showed slightly hypercellular bone marrow with bone marrow fragments and trilinear engraftment, with less than 1% blast cells. Unseparated donor chimerism > 99%. The patient has had no complications following her second allo‐HSCT and has satisfactory graft function and no signs of GvHD or relapse of either SM or AML on Day +90. She is currently undergoing regular follow‐up consultations with bone marrow biopsies each third month of the first year, in addition to imaging when needed. Follow‐up imaging thus far has not shown osteolytic‐appearing lesions.

## 3. Discussion

This case report describes a patient who was concurrently diagnosed with AML and SM, who underwent allo‐HSCT with curative intent. While the efficacy of allo‐HSCT in AML is well established—primarily attributed to the graft‐versus‐leukemia (GVL) effect mediated by donor T‐lymphocytes—its impact on SM remains unclear [9, 10]. Notably, both diseases showed complete remission immediately post‐transplant, prompting the question of why AML relapsed in the absence of SM recurrence. The GVL effect is a well‐documented phenomenon in which the immune system, particularly donor T‐cells, attacks leukemia cells, thus contributing to remission and improved outcomes [11]. However, this immune response is not as robust against SM. It has been suggested that mast cells in SM may be less susceptible to immune‐mediated elimination compared with leukemia cells. On the contrary, mast cells may be more vulnerable to cytotoxic therapy as well as to midostaurin treatment [12]. This differential response could be attributed to various factors, such as the relative resistance of mast cells to graft‐versus‐host immunity or differences in the immunogenicity of the two diseases.

Relapse of AML post‐allo‐HSCT without concurrent SM recurrence is rare but not entirely unprecedented. In fact, some studies have shown that neoplastic mast cells and myeloid leukemic blasts share a common hematopoietic progenitor, i.e., they arise from the same mutated progenitor cell [13]. Therefore, we would expect re‐emergence of SM concomitantly to AML.

While relapse in AML patients is common, nearly 43% of relapses occur within six months post‐transplant [10]. In these cases, the likelihood of relapse diminishes over time, with a median relapse time of seven months post‐transplant [10]. Interestingly, clonal development plays a crucial role in relapse. In most patients, relapse occurs due to the survival and expansion of a leukemic clone that evades the immune system [14]. However, SM cells, which originate from a different clonal lineage, do not appear to re‐emerge in this patient. One potential explanation could be the different mutational landscape or microenvironmental factors that influence the survival of SM mast cells versus AML leukemic cells, potentially limiting the recurrence of SM even in the context of an AML relapse [3].

In general, it is uncommon for relapses to occur as late as it did in this patient. Late relapses, occurring after the first year or more post‐transplant, are unusual in AML patients, as most relapses occur within one year post transplant [10]. The long duration of remission before relapsing in this case is exceptional, suggesting that the patient experienced a prolonged period of immune surveillance and control. However, studies have shown that the post‐transplant remission duration is a key predictor of long‐term survival after relapse. According to Webster et al. and data from the Center for International Bone Marrow Transplant Registry (CIBMTR), the 3‐year overall survival rate drops to only 4% among patients who relapse within six months, illustrating the dismal prognosis associated with early relapse [10].

Two primary immunological mechanisms are thought to contribute to relapse after allo‐HSCT, T‐cell exhaustion and downregulation of HLA Class II on leukemic blast cells [15]. These mechanisms could explain why some patients experience relapses despite ongoing GVHD, which, in most cases, is protective against leukemia recurrence. However, in rare cases, relapses occur despite the presence of GVHD, and patients who relapse despite GVHD tend to have poorer survival outcomes [10].

Currently, there is limited literature on AML relapse in patients with concomitant systemic mastocytosis. In SM, mast cells originate from pluripotent bone marrow progenitor cells expressing the CD34 antigen. In healthy individuals, mast cell proliferation is tightly regulated by cytokines and c‐kit proto‐oncogene receptor signaling [16, 17]. However, the mechanisms driving mast cell proliferation and accumulation in mastocytosis are yet to be fully understood [17]. Treatment typically aims to inhibit mast cell degranulation through both IgE‐mediated and IgE‐independent pathways [18]. Notably, in this case, the patient showed no evidence of SM recurrence following transplantation.

Our case presentation raises an important question; why did our patient relapse with AML without exhibiting signs of SM recurrence post‐transplant? The differential immune responses to AML and SM, the possibility of distinct clonal development pathways for the two diseases, and the rarity of late relapses all contribute to the complexity of understanding this patient’s outcome. Both at diagnosis and at relapse, the tumor cells retain the same myelodysplasia‐associated mutations (*RUNX1*, *BCOR*, and *ASXL1*), pointing to a shared clonal ancestry. In contrast, the KIT D816V mutation—implicated in ongoing *KIT* activation and mast‐cell maturation—appears only at the time of diagnosis, while *RAS* mutations arise solely at relapse. This shift in the mutational landscape may underlie the altered disease phenotype.

Further research and patient characteristics are needed to better understand the rare disease entity of AML with SM and to optimize and improve treatment outcomes for this patient cohort.

All authors have read and approved the final version of the manuscript. Sona Varadnyan has full access to all of the data in this study and takes complete responsibility for the integrity of the data and the accuracy of the data analysis [19].

## Funding

No funding was received for this manuscript.

## Conflicts of Interest

The authors declare no conflicts of interest.

## Data Availability

The data that support the findings of this study are available from the corresponding author upon reasonable request.
